# Green Polyurethanes from Renewable Isocyanates and Biobased White Dextrins

**DOI:** 10.3390/polym11020256

**Published:** 2019-02-03

**Authors:** Jakob Konieczny, Katja Loos

**Affiliations:** 1 Macromolecular Chemistry and New Polymeric Materials, Zernike Institute for Advanced Materials, University of Groningen, Nijenborgh 4, 9747 AG Groningen, The Netherlands; jkonieczny845@gmail.com; 2 Dutch Polymer Institute (DPI), P.O. Box 902, 5600 AX Eindhoven, The Netherlands

**Keywords:** starch, carbohydrates, polyurethanes, film formation, renewable resources

## Abstract

Polyurethanes (PUs) are an important class of polymers due to their low density and thermal conductivity combined with their interesting mechanical properties—they are extensively used as thermal and sound insulators, as well as structural and comfort materials. Despite the broad range of applications, the production of PUs is still highly petroleum-dependent. The use of carbohydrates in PU synthesis has not yet been studied extensively, even though, as multihydroxyl compounds, they can easily serve as crosslinkers in PU synthesis. Partially or potentially biobased di-, tri- or poly-isocyanates can further be used to increase the renewable content of PUs. In our research, PU films could be easily produced using two bio-based isocyanates—ethyl ester L-lysine diisocyanate (LLDI] and ethyl ester l-lysine triisocyanate (LLTI)—, one commercial isocyanate—isophorone diisocyanate (IPDI), and a bio-based white dextrin (AVEDEX W80) as a crosslinker. The thermal and mechanical properties are evaluated and compared as well as the stability against solvents.

## 1. Introduction

Polyurethanes (PUs) are versatile polymers, created from the reaction between a polyol and a polyisocyanate, that are used in a large range of applications, such as automotive, furniture, construction, footwear, etc. The PU industry is strongly dependent on fossil-based polyols and polyisocyanates. Developing novel sustainable polyols from valuable biobased building blocks is a first step toward strong and durable development [1,2,3].

In recent years, the preparation of PUs from renewable sources such as vegetable oil-based materials [4], lignin [5], limonene [6], and even coffee grounds [7] has been receiving increasing attention because of economic and environmental concerns [8,9,10,11,12,13]. The use of carbohydrates in PU synthesis has not yet been studied extensively, even though, as multihydroxyl compounds [14,15,16,17,18,19], they can easily serve as crosslinkers in PU synthesis. Furthermore, they can impart mechanical strength, biodegradability, and biocompatibility to the produced PUs. Carbohydrates are reported to be embedded in PU networks by using them as composites/fillers and by covalent linkage with isocyanate to form crosslinked networks [20]. Solanki et al., for instance, synthesized castor oil-based PUs crosslinked with starch and reported excellent mechanical properties of the produced materials [21]. Carbohydrates, such as cellulose [22,23,24,25,26] or starch [27] nanocrystals, have been used as useful fillers in PUs. We have recently reported PU synthesis and film formation capacity using an asymmetric cyclic aliphatic diisocyanate and acetylated and pristine partially hydrolyzed amylopectin/white dextrin (AVEDEX W80) as a crosslinker [28,29].

The next logical step towards a greener PU formulation is then, of course, the use of partially or potentially biobased di-, tri- or poly-isocyanates. While synthetic pathways to biobased isocyanates still require phosgene as a petroleum-based reagent, several commercial isocyanates with high renewable content exist, such as isocyanates based on fatty acids or amino acids.

l-lysine-based diisocyanates have frequently been used for biomedical PUs in the last several years as drug delivery systems, hydrogels, implant materials, etc. [30,31,32,33,34,35,36]. In some very inspiring recent research, the L-lysine based ethyl ester l-lysine diisocyanate was compared to petrol-based hexamethylene diisocyanate, and isophorone diisocyanate in terms of reactivity and final properties of the PU [37,38,39,40,41] and even fully renewable thermoplastic poly(ester urethane urea)s were produced [42]. More studies on enzymatic breakdown/biodegradability of (biobased) PUs are conducted in recent years proving the importance of environmentally friendly PU applications [9,43,44,45,46,47].

In our previous work, we found that pristine AVEDEX W80 resulted in the best PU films [29,48] (i.e., with no cracks, inhomogeneity, or swelling) and we, therefore, continue with this system and two bio-based isocyanates—ethyl ester l-lysine diisocyanate (LLDI) and ethyl ester l-lysine triisocyanate (LLTI)—and one commercial isocyanate—isophorone diisocyanate (IPDI). The optimal reaction conditions in terms of casting time and temperature, isocyanate used, amount of solvent, and excess of isocyanate were also determined.

## 2. Materials and Methods

Already degraded amylopectin–white dextrin (AVEDEX W80) was obtained from AVEBE (Veendam, The Netherlands) and used without further purification. Iodine was provided by Boom and purified by sublimation twice. Isophorone diisocyanate (IPDI) was provided by Merck (Darmstadt, Germany) and ethyl ester L-lysine diisocyanate (LLDI) and ethyl ester l-lysine triisocyanate (LLTI) were provided by Shanghai Infine Chemicals Co. Ltd. (Shanghai, China). Acetic anhydride ((CH_3_CO)_2_O) (≥99.0%) and propionic anhydride ((CH_3_CH_2_CO)_2_O) (≥96.0%) were provided by Fluka (Bucharest, Romania), sodium thiosulfate (NaS_2_O_3_) (≥98.0%) and DMSO (99%) were provided by Sigma-Aldrich (St. Louis, MO, USA) and ethanol (≥99%) was purchased from Merck (Darmstadt, Germany) and used without further purification.

Attenuated total reflection-Fourier transform infrared (ATR-FTIR) measurements were characterized by a Bruker IFS88 FT-IR spectrometer (Leiderdorp, The Netherlands). For each sample, 128 scans were performed.

Thermal transitions were measured by differential scanning calorimetry (DSC) on a TA-Instruments Q1000 (Hertfordshire, UK). The heating and cooling rates were 10 °C/min. To remove the remaining water and solvents in the polymer, the tested sample was first heated to 100 °C at 10 °C/min, maintained at this temperature for 5 min, and then cooled to room temperature before the standard DSC measurement. Each sample was run for 4 cycles, and the T_g_ was calculated from the second.

Specimens were made from PU samples and were tested on a tensile tester to study their mechanical properties, such as stress–strain behavior and Young’s modulus. The samples were kept between two Teflon plates to ensure a smooth and straight sample form, especially samples that had the tendency to break easily. Mechanical properties were tested on an Instron Tensile Tester (Boechout, Belgium) with a pulling speed of 10 mm/min. The distance between the clamps was 25 mm with a pressure of 7 bar.

### 2.1. Crosslinking of Acylated Polyols with Di- and Tri-Iisocyanates

Point two gram polyol AVEDEX W80 was dissolved in 7.5 mL of solvent (DMSO). A 1.05-fold excess of a di- and/or tri-isocyanate (i.e., a 1.05-fold excess of –NCO groups to –OH groups) was added, the mixture was stirred for 1 to 2 min, and the resulting homogenous mixture was poured onto a petri dish. The petri dish was placed on a heating block at a defined temperature (120 °C), covered with filter paper and the heating block was sealed to provide a solvent atmosphere. After a defined period of time, the petri dish was removed from the heating block.

### 2.2. Resistance against Organic Solvents

To investigate the resistance of the obtained polyurethane films against organic solvents, samples were weighed, after drying to constant weight, before and after immersing them in an organic solvent.

## 3. Results

**Scheme 1:**
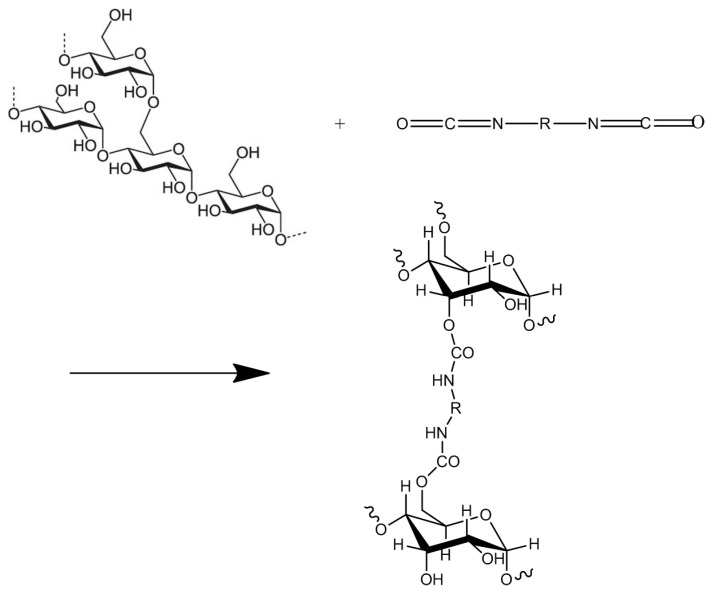
Reaction scheme of the crosslinking of carbohydrates as polyol components with isocyanates.

The synthesis of polyurethane films by the crosslinking of carbohydrates as polyol components with isocyanates—see Scheme 1—was successfully conducted. The selection of isocyanates available for PU synthesis with polysaccharide polyols is almost unrestricted. As aromatic isocyanates might introduce a color into the final material, we did not consider them here. Two bio-based isocyanates—ethyl ester l-lysine diisocyanate (LLDI) and ethyl ester l-lysine triisocyanate (LLTI)—and one commercial isocyanate—isophorone diisocyanate (IPDI)—were used.

PUs derived from the diisocyanates IPDI and LLDI were too hard and brittle to test after drying. LLTI-based PUs behaved more like a rubber than a plastic; after drying the material became hard, but it was still flexible. PUs from IPDI always resulted in a hard and brittle material (after drying), but they are the only ones that are transparent due to the yellow color of the biobased isocyanates. PUs from LLDI also became brittle after drying, but the material was soft and easy to scratch.

**Figure 1:**
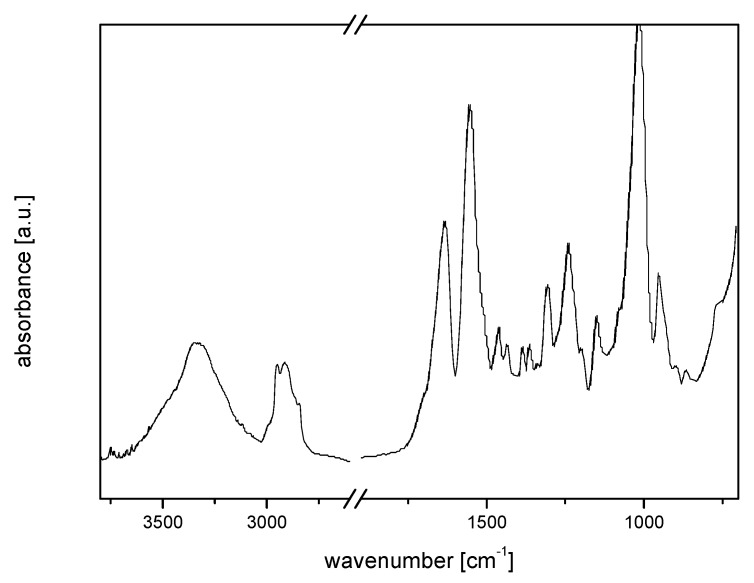
Fourier transform infrared (FT-IR) spectrum of isophorone diisocyanate (IPDI) based polyurethanes (PU) (75% IPDI/25% ethyl ester l-lysine triisocyanate (LLTI)).

FT-IR spectra of all films show successful PU synthesis; Figure 1 shows the FT-IR spectrum of an IPDI-based PU. The peaks of the amide I and amide II bands from the newly formed polyurethane linkages are visible at 1550 cm^−1^ and 1650 cm^−1^ respectively. All materials are not fully reacted, i.e., not all of the OH-functions of the polyol formed urethane linkages with the di- and tri-isocyanates, not even while using a 50% excess of isocyanate. It seems to be impossible to convert all free OH groups in pristine AVEDEX W80 as crosslinking becomes too high. In our previous work, we also observed that PU films with an excessively high crosslinking become brittle so that a full conversion is not desirable from the start.

To improve the properties of synthesized PUs, we decided to perform experiments with mixtures of isocyanates from this series. Two series of experiments with different ratios of two isocyanates were carried out, with the diisocyanate always the main component of the isocyanate and up to 50% (by volume) LLTI as a “co”-isocyanate. Combinations of the two diisocyanates were also prepared, with different ratios of these two isocyanates.

**Figure 2:**
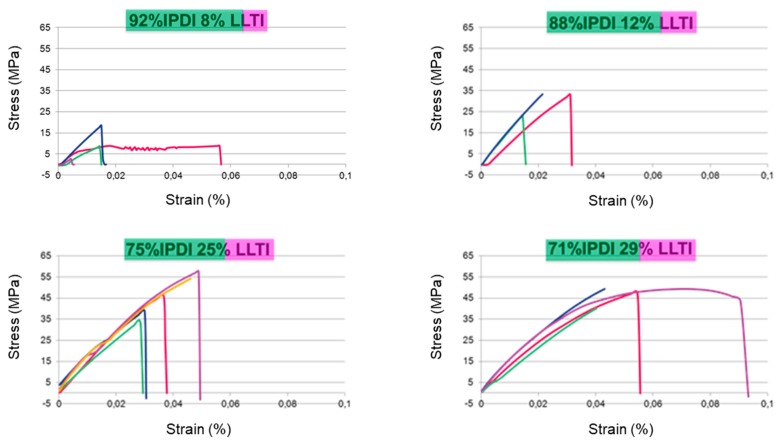
Stress–strain behavior of PUs with different IPDI/LLTI ratios.

**Figure 3:**
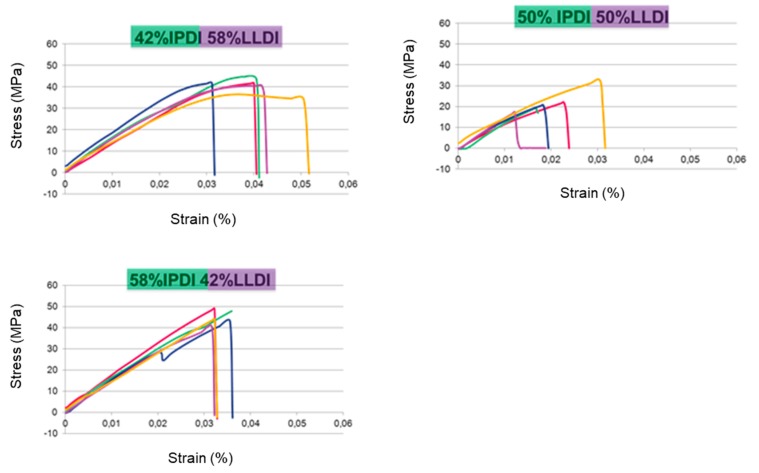
Stress–strain behavior of PUs with different IPDI/ ethyl ester L-lysine diisocyanate (LLDI) ratios.

**Figure 4:**
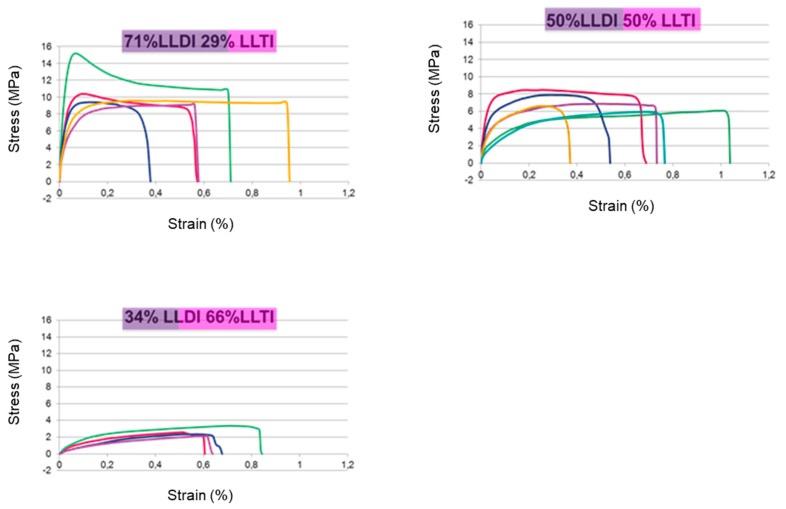
Stress–strain behavior of PUs with different LLDI/LLTI ratios.

Mechanical properties of the produced films were tested on an Instron Tensile Tester with a pulling speed of 10 mm/min and are summarized in Table 1—the stress–strain curves are shown in Figure 2, Figure 3 and Figure 4.

Gradual replacement of the hard diisocyanate with the soft triisocyanate resulted in a softer, less rigid and breakable material. At 29% LLTI, the strain at maximum load was twice as large as at 12% LLTI and samples were much more flexible. The curve progression was straight at 8% LLTI, but at 29% LLTI the curve already looked much more like a stress–strain curve from a rubber, with a plateau at its maximum and much higher strain than at 8% LLTI (see Figure 2).

Shifting the ratio of two diisocyanates slightly from a 1:1 ratio toward either side did not change the mechanical properties of the material. As seen in Table 1, neither the average modulus, nor the stress or the strain at max load change significantly. Only the curve progression becomes more rubbery, like in the sample with 58% LLDI.

Gradually replacing LLDI with LLTI during solvent casting resulted in a much harder material compared to PUs consisting only of LLDI. At approximately 30% LLTI, the material has the most desirable properties, as the surface is hard, but the whole sample is still flexible enough to bend, and the strain before breaking is over 100%. The LLDI/LLTI PU is by far the one with the greatest strain at maximum load (Table 1).

Increasing the percentage of LLTI changes the PU properties even further, with the flexibility almost lost and the stress the sample can withstand before breaking very low compared with the other combinations of isocyanates. The maximum strain is also greatly reduced with an increase of LLTI above 30%.

**Figure 5:**
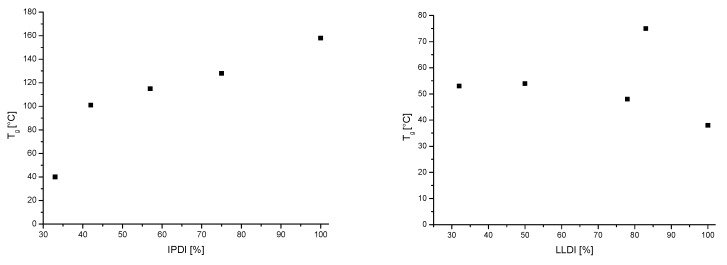
Relationship between T_g_ and isocyanate ratio of IPDI-based (**left**) and LLDI-based (**right**) PU films.

The thermal properties of the produced PUs were assessed by differential scanning calorimetry. The highest T_g_ was observed for the IPDI-based PU (T_g_ = 157 °C). To investigate the dependence of T_g_ on the ratio of isocyanates, samples with different ratios were tested; this data is presented in Figure 5. In the case of the IPDI-based PUs. the effect is clearly visible; lowering the IPDI ratio lowers the T_g_. In the case of the LLDI/LLTI-based PUs, change of the LLDI/LLTI ratio does not result in significant change in T_g_; increasing the percentage of LLDI results in T_g_ being slightly shifted towards lower temperatures. At this point, we cannot explain the one outlier at around 80% LLDI content.

To test the resistance of the PU films against organic solvents, different samples were chosen and immersed in DMSO for 24 h. DMSO was chosen because it showed the most aggressive behavior toward the films. The difference in weight before and after immersion after drying until constant weight was calculated; this data is presented in Table 2. As observed by FT-IR measurements, the samples are not changed chemically during the solvent resistance test, indicating that the weight loss mainly results from washing away of unreacted polyol from the PU sample (as indicated by the lower OH-peak and higher amide I and II peaks). Samples made with IPDI seem to be more resistant against attacks from DMSO. The maximum weight loss in samples with IPDI is 7%, as compared to 12.5% for LLTI and almost 30% for LLDI.

## 4. Conclusions

Unmodified AVEDEX W80 was able to form homogenous films with the tested di- and tri-isocyanates, after optimization of several reaction conditions. With commercially used IPDI, the resulting material is transparent, rigid, and tends to break quite easily, especially after drying in the vacuum oven.

PUs derived from LLDI are flexible, but rather milky than transparent and they experience a coarse surface. They are more flexible than the IPDI based PUs but also tend to crack when fully dry as proven by tensile tests. As the biobased LLDI and LLTI are colored, the resulting films have a slightly yellow color (more pronounced in the PU films of LLTI).

Addition of the trifunctional isocyanate LLTI to either IPDI- or LLDI-based formulations resulted in a dry material, which has a high scratch resistance but is still flexible without breaking. Ratios of the isocyanates play an important role in the final properties of the material.

In general, the IPDI-based PUs shows higher stability in DMSO than the biobased. Additionally, the biobased PUs tend to swell in organic solvents, which is not the case for IPDI-based PUs. Besides differences in brittleness, differences in T_g_ can be observed in all four PUs. Combining two of these isocyanates results in changes in T_g_, especially while replacing IPDI with LLDI, resulting in a drop in T_g_. Gradually replacing LLDI with LLTI does not result in significant T_g_ changes in the material.

The mechanical properties of these materials depend on the isocyanate used for the PU formulation. If IPDI is the major component in the isocyanate mixture, the mechanical behavior is that of a hard and brittle material. Addition of the trifunctional isocyanate results in a much more rubbery material, visible in the stress–strain diagram. The slope of the curve starts to decrease at some point, reaching a plateau, which then leads to “necking” in the tensile tests.

## Figures and Tables

**Table 1:** Average Young’s modulus, maximum stress and maximum strain of PUs with different isocyanate ratios.

% LLTI	% IPDI	Young’s Modulus (MPa)	Stress at Max Load (MPa)	% Strain at Max Load
8	92	1126	9	2
12	88	1536	33	2
25	75	1554	44	3
29	71	1427	46	5
**% LLDI**	**% IPDI**			
42	58	1600	44	3
50	50	1501	22	2
58	42	1585	41	3
**% LLDI**	**% LLTI**			
34	66	22	2	60
50	50	195	6	92
66	34	18	2	123

**Table 2:** Weight loss of different ethyl ester polyurethanes (PU) samples after immersion in DMSO for 1d.

isocyanate	IPDI	LLDI	LLDI	LLTI	LLDI/LLTI	IPDI/LLTI	LLDI/LLTI	IPDI/LLTI	LLDI/LLTI	LLDI/LLTI	LLDI/LLTI
ratio	100	100	100	100	50/50	66/33	66/33	75/25	71/29	92/8	79/21
weight before (g)	0.0582	0.0750	0.0713	0.1223	0.0812	0.0578	0.1060	0.0474	0.0955	0.1033	0.1335
weight after (g)	0.0541	0.0530	0.0650	0.1350	0.0809	0.0547	0.0927	0.0442	0.0948	0.0970	0.1233
difference %	−7.04%	−29.33%	−8.84%	10.38%	−0.37%	−5.36%	−12.55%	−6.75%	−0.73%	−6.10%	−7.64%

## Abbreviations

PU	polyurethane
AVEDEX W80	amylopectin/white dextrin
LLDI	ethyl ester l-lysine diisocyanate
LLTI	ethyl ester l-lysine triisocyanate
IDPI	isophorone diisocyanate
